# A Case of Vaccine-Induced Thrombocytopenic Thrombosis Manifesting as Cerebral Venous Thrombosis and Intracerebral Bleed

**DOI:** 10.7759/cureus.33318

**Published:** 2023-01-03

**Authors:** Shannay E Bellamy, Brian A Loor

**Affiliations:** 1 Internal Medicine, Jersey City Medical Center, Jersey City, USA

**Keywords:** anti-platelet factor 4 (apf-4) antibodies, vaccination, covid19, cerebral venous sinus thrombosis, vaccine-induced thrombotic thrombocytopenia

## Abstract

Vaccine-induced immune thrombotic thrombocytopenia (VITT) is a rare but detrimental syndrome that has been most commonly reported after the administration of vaccination for the prevention of viral infections. VITT often presents with thrombosis at unusual sites such as cerebral venous sinuses, portal, splanchnic or hepatic veins, in association with thrombocytopenia and elevated anti-platelet factor 4 (aPF-4) antibodies. We describe the case of a young male patient who developed thrombocytopenia, cerebral sinus venous thrombosis, and intracerebral bleed 12 days after receiving the Ad26.COV2.S (Janssen/Johnson&Johnson) COVID-19 vaccine.

## Introduction

The COVID-19 pandemic resulted in enormous health care and economic burden worldwide. The morbidity and mortality of this viral pandemic resulted in massive efforts for the timely development and distribution of vaccinations against this virus. Several vaccinations against COVID-19 were developed to reduce the impact of this global pandemic, and with worldwide distributions of these vaccinations worldwide, several common and rare adverse effects of vaccinations have been reported. One rare but potentially life-threatening reported complication of COVID-19 vaccination is vaccine-induced immune thrombotic thrombocytopenia (VITT). VITT is an uncommon prothrombotic syndrome that presents with a constellation of findings that includes venous or arterial thrombosis, mild to severe thrombocytopenia, and anti-platelet factor 4 (aPF-4) antibodies [1]. VITT was first described as a rare adverse effect associated with the ChAdOx1 nCoV-19 (AstraZeneca vaccine) and then subsequently with the Ad26.COV2.S (Janssen/Johnson&Johnson) vaccine [2]. Individuals that developed this rare vaccine-induced complication were previously healthy, and very few had any known personal or family history of hypercoagulable states. Clinical manifestations of VITT became apparent five to 24 days after the administration of the vaccine [1]. These clinical manifestations included thrombus formation at unusual sites such as cerebral venous sinuses, portal, splanchnic or hepatic veins. Arterial thromboses were also reported, including cases of pulmonary embolism [3]. Laboratory findings included mild to severe thrombocytopenia and the occurrence of anti-platelet factor 4 (aPF-4) antibodies in the absence of heparin exposure. Here, we present a case of a 33-year-old, otherwise healthy male who presented with VITT 12 days after receiving the Ad26.COV2.S (Janssen/Johnson&Johnson) vaccine.

## Case presentation

We present the case of a 33-year-old Caucasian male with no known prior medical illness and no significant family or social history who was brought to the emergency department after a witnessed episode of first-time generalized tonic-clonic seizure activity associated with a fall and facial trauma.

Twelve days prior to this presentation, the patient had been vaccinated with Ad26.COV2.S (Janssen/Johnson&Johnson) vaccine for COVID-19. He had received one dose of the vaccine. He had been reported to be in good health prior, with no prior illnesses, hospitalization, and no prescribed drug or recreational drug use. One week after receiving the vaccination, the patient experienced daily occipital headaches, minimally relieved by acetaminophen. At that time, he denied any fevers, chills, visual changes, neck stiffness, or any focal motor or sensory neurological deficits.

On presentation to the emergency room, he had a blood pressure of 161/112mmHg, heart rate of 98 bpm, temperature of 97.9 F, respiratory rate of 18/min, and oxygen saturation of 98% on room air. On physical examination, he was awake and alert and following commands appropriately but was disoriented to place and time. He was noted to have a superficial laceration to the forehead. Further neurological examination was within normal limits, and examination of other systems was unremarkable.

Laboratory investigations were significant for thrombocytopenia with platelets of 74 K/UL, mildly elevated prothrombin time of 16 seconds, and significantly elevated d-dimer of 3117 DDu ng/ml (see Table 1). SARS-CoV-2 rapid antigen, COVID-19 antigen test, influenza A and B, and human immunodeficiency virus (HIV) tests were all negative.

**Table 1:** Laboratory test results on admission Hb - hemoglobin; MCV - mean corpuscular volume; WBC - white blood cell count; PT - prothrombin time; INR - international normalized ratio; PTT - activated partial thromboplastin time; Na - sodium; K - potassium; Cl - chloride; CO_2_ - serum bicarbonate; BUN - blood urea nitrogen; Ca - calcium; AST - aspartate aminotransferase; ALT - alanine aminotransferase; ALP - alkaline phosphatase

Laboratory test on admission	Result	Normal range
Hb	15.3 g/dl	14-18g/dl
MCV	91.8 fl	80.0-100.0 fl
Platelets	74 K/UL	130 – 400K/UL
WBC	11.6 K/UL	4.5-11.0K/UL
PT	16 sec	12-15.1sec
INR	1.23	0.85-1.14
PTT	28 sec	25.4-36.7 sec
Fibrinogen	258 mg/dl	244-550 mg/dl
D-dimer	3117 DDu ng/ml	<243 DDu ng/ml
Na	143 mmol/L	136-145 mmol/L
K	3.8 mmol/L	3.5-5.1 mmol/L
Cl	107 mmol/L	98-107 mmol/L
CO_2_	22 mmol/L	20-31 mmol/L
BUN	10 mg/dl	9-23 mg/dl
Creatinine	1.05 mg/dl	0.70-1.30 mg/dl
Ca	9.5 mg/dl	8.7-10.4 mg/dl
AST	26 Units/L	8-34 Units/L
ALT	29 Units/L	10-49 Unis/L
ALP	67 Units/L	46-116 Units/L

**Figure 1:**
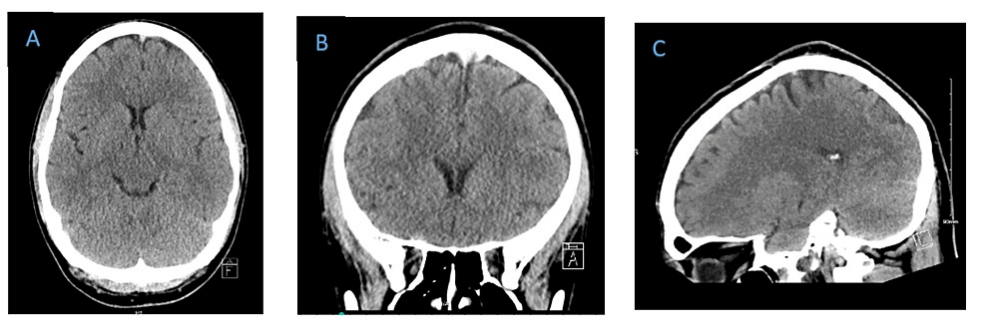
CT of the head done on admission A: axial view; B: coronal view; C: sagittal view

CT scan of the head without contrast done at the time of presentation was unremarkable (Figure 1).

**Figure 2:**
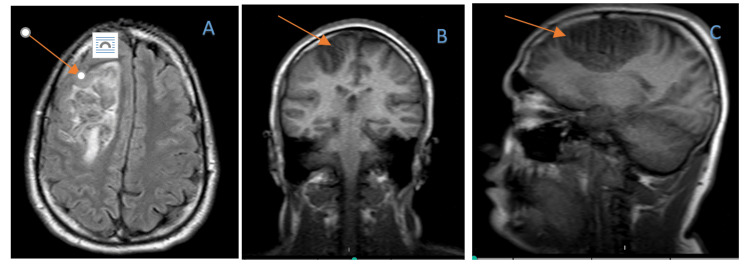
MRI of the brain showing the area of signal abnormality in the frontal lobe with regional mass effect with concern for hemorrhagic infarct A: axial view; B: coronal view; C: sagittal view

While awaiting further evaluation in the emergency room, the patient had another episode of witnessed generalized tonic-clonic seizure activity while in bed, which aborted after a total of 2mg of intravenous lorazepam and 5mg of midazolam. He was subsequently noted to be post-ictal and drowsy and was taken for an urgent MRI of the brain. MRI of the brain showed a new, evident, fairly sizable area of signal abnormality in the frontal lobe with region mass effect suspicious for a hemorrhagic infarct related to thrombosis of the regional cortical vessels (Figure 2).

**Figure 3:**
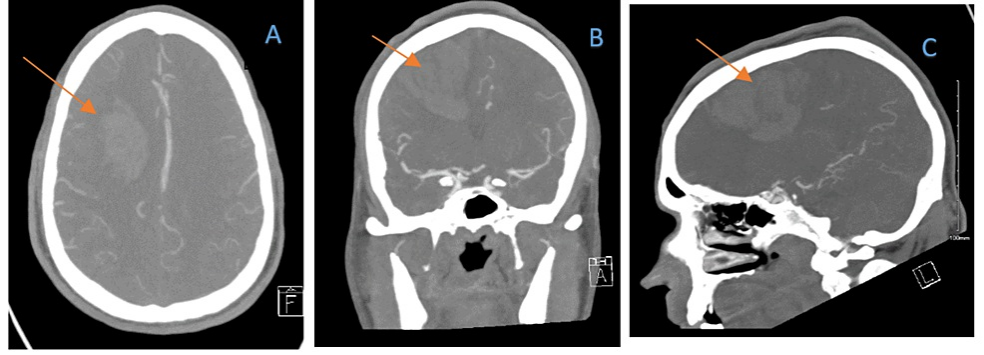
CT angiogram of the head showing sizable parenchymal hemorrhage in the right frontal lobe in the setting of thrombosis of the superior sagittal, torcular herophili, and right transverse venous sinuses A: axial view; B: coronal view; C: sagittal view

CT angiogram of the head showed near total occlusive dural venous sinus thrombosis in the superior sagittal sinus, torcular herophili, and right transverse sinus, as well as some right-sided cortical veins in the vertex. It also showed a sizable parenchymal hemorrhage in the right frontal lobe with a hemorrhagic infarct (Figure 3).

He was then loaded with IV levetiracetam for seizure activity. Neurology and neurosurgical teams were both consulted.

Given these clinical findings of cerebral venous sinus thrombosis (CVST) associated with thrombocytopenia in a healthy young male recently receiving a COVID-19 vaccination, differential diagnoses, including possible underlying rheumatological disease and vaccine-induced thrombocytopenia, were made. Further hematological and immunological laboratory investigations were sent. Antinuclear antibody test, lupus anticoagulant, and anticardiolipin antibodies were negative. Factor II, V, VII, and X levels were within normal limits. Serum protein electrophoresis showed a normal pattern with no monoclonal bands and serum immunoglobulin A, G, and M. However, heparin-induced anti-platelet factor 4 (aPF-4) antibodies were positive. He was admitted to the medical intensive care and was commenced on intravenous immunoglobulin therapy. He was noted to have a progressive decline in neurological status, requiring intubation for airway protection and mechanical ventilation. Subsequent CT brain imaging revealed worsening intracerebral edema with associated midline shift, and he was taken for hemicraniectomy and thrombectomy. After surgery, he was commenced on argatroban. Despite surgical intervention, the patient had no further improvement in neurological status. A video electroencephalogram (EEG) was done to rule out the possibility of underlying non-convulsive seizure activity for his minimal improvement in neurological status, but it did not show any evidence of seizure activity. Video EEG did show continuous right hemispheric slowing of brain activity consistent with cortical dysfunction. Unfortunately, this patient was unable to be weaned from mechanical ventilation and subsequently had placement of tracheostomy and percutaneous endoscopic gastrostomy tube. He was then discharged to a long-term care facility for further management.

## Discussion

The COVID-19 pandemic caused by SARS-CoV-2 led to expedited development and production of vaccinations to limit the spread of the virus [1]. Four vaccinations to prevent the spread of the SARS-CoV-2 virus were then approved by European Medicines Agencies by March 2021, including the two messenger RNA vaccines - mRNA-1273 (Moderna) and the BNT162b2 (Pfizer-BioNTech) vaccines; and two adenoviral vaccines - ChAdOx1 nCov-19 (AstraZeneca) and Ad26.COV2.S (Janssen/Johnson&Johnson)[2].

Vaccine-induced thrombocytopenia thrombosis (VITT) describes a rare clinical syndrome involving both venous and arterial thromboses, thrombocytopenia, and positive anti-platelet factor 4 (aPF-4) antibodies.

aPF-4 antibodies are most commonly found in the setting of heparin-induced thrombocytopenia, where the heparin- aPF-4 antibody complexes bind to and activate platelets, resulting in a hypercoagulable condition that leads to arterial and/or venous thrombus formation [4]. However, in the cases of VITT, these aPF-4 antibodies are often found in high titer in the absence of exposure to heparin or heparin-derived products. The exact mechanism for the development of these aPF-4 antibodies in VITT is still unclear but is thought to be related to one or more components of the vaccine, which are still yet to be determined [5].

Initial symptoms of VITT typically occur six to 14 days after administration of the adenoviral vaccines. Venous and arterial thrombosis have been the most prominent clinical manifestations of VITT, with cerebral venous sinus thrombosis (CVST) being the most common and clinically severe form of thrombosis. CVST occurring in VITT often involves multiple cerebral venous sinuses, with intracerebral hemorrhage occurring secondary to increased intravenous pressure caused by the obstruction of venous outflow in the setting of thrombocytopenia [6], which is consistent with the findings found in our patient. 

Since VITT is a relatively new syndrome, with the exact pathophysiology remaining unclear, there has been no clear guideline set forth for its management. Given its close pathophysiologic similarities to heparin-induced thrombocytopenia, most treatment recommendations have been established based on the extrapolation of data from heparin-induced thrombocytopenia. Intravenous immunoglobulin and anticoagulation with non-heparin-derived anticoagulants are the mainstays of treatment of VITT [7]. Intravenous immunoglobulin therapy is thought to decrease the formation of aPF-4 antibodies and decrease the activation of platelets. Non-heparin anticoagulants are used to decrease the risk of the formation of new thrombi and decrease the risk of propagation of existing thrombi and should be started in patients with suspected and confirmed VITT. Anticoagulants that are thought to be safe for use in VITT include direct thrombin inhibitors (e.g., argatroban, dabigatran), oral factor Xa inhibitors (e.g., apixaban, rivaroxaban), and fondaparinux [8]. It is recommended that platelet infusions be avoided as they can result in an increased risk of further thrombosis [9].

The clinical course and treatment described in our case show similarities to numerous other reported cases of VITT associated with adenoviral COVID-19 vaccinations. One such case is reported by Szypowski et al., where a healthy young male presented with first-time generalized seizure activity seven days after receiving the ChAdOx1 nCov-19 (AstraZeneca) and brain imaging revealing extensive CVST and cerebral hemorrhage. Similar to the case presented here, the patient described in that case had a deterioration in neurological status requiring intubation and mechanical ventilation, and in both cases, treatment consisted of intravenous immunoglobulin and non-heparin anticoagulation. The difference identified was the type of non-heparin anticoagulation used, with our patient receiving argatroban and another patient receiving fondaparinux [10]. Despite the implementation of these recommended first-line therapies, both patients showed no significant neurologic improvement.

In some reported cases, glucocorticoids are used in the initial management of VITT, along with intravenous immunoglobulin and non-heparin anticoagulants. Guidelines, including the International Society for Thrombosis and Haemostasis (ISTH) and the British Society of Haematology (BSH), suggest that glucocorticoids may be helpful for the management of VITT, although this has not clearly been proven [11,12]. The initiation of glucocorticoid therapy was not done in our case but could have been considered as a part of the initial management regimen. 

For patients like the one presented here, where there is little clinical improvement with the initial first-line therapies, including glucocorticoids, the subsequent treatment remains unclear. In these cases of VITT deemed refractory to the initial management, plasmapheresis may be considered as an alternative therapy [13]. The mechanism of plasmapheresis in VITT involves the removal of the aPF-4 antibodies thought to be responsible for thrombosis. In a case series by Patriquin et al., which describes three cases of VITT due to the ChAdOx1 nCov-19 (AstraZeneca) vaccine, plasmapheresis was commenced as salvage therapy after initial treatment with intravenous immunoglobulin, glucocorticoids and non-heparin anticoagulation did not result in any significant clinical improvement or improvement in platelet counts. In each of these cases, plasmapheresis was initiated within the first six days of presentation, with subsequent clinical improvement [13]. In a case of VITT reported by Major et al., initially refractory to treatment with intravenous immunoglobulin, intravenous and oral glucocorticoids, and non-heparin anticoagulation, significant clinical improvement was observed after the initiation of plasmapheresis, even though it was started more than thirty days after initial presentation [14]. These two clinical reports suggest that the plasmapheresis may be potentially beneficial as salvage therapy when used both early and later in the course of the presentation of refractory VITT.

## Conclusions

Our patient presented with CVST complicated by intracerebral hemorrhage, a reported serious adverse effect of VITT caused by the administration of the Ad26.COV2.S (Janssen/Johnson and Johnson) COVID-19 vaccine. Despite the use of recommended medical treatment and appropriate surgical intervention, our patient suffered significant morbidity. Plasmapheresis may be considered a therapeutic option for cases that are unresponsive to the initial recommended therapy for VITT, including intravenous immunoglobulin, glucocorticoids, and non-heparin anticoagulation.

Although this case outlines an unfavorable outcome, it is important to remember that the incidence of VITT remains low, and the benefit of vaccination against COVID-19 continues to outweigh the risks.
