# Correction: A Case of Vaccine-Induced Thrombocytopenic Thrombosis Manifesting as Cerebral Venous Thrombosis and Intracerebral Bleed

**DOI:** 10.7759/cureus.c110

**Published:** 2023-04-24

**Authors:** Shannay E Bellamy, Brian A Loor

**Affiliations:** 1 Internal Medicine, Jersey City Medical Center, Jersey City, USA

This article has been corrected as follows: The Ad26.COV2.S (Janssen/Johnson and Johnson) vaccine was incorrectly referred to as an mRNA vaccine multiple times when it is in fact an adenoviral vector vaccine. All inaccurate statements describing the vaccine as an mRNA vaccine have been corrected. In addition, the authors have elected to add an additional sentence stating that the patient received one dose of the vaccine, as this was omitted from the original version.

